# Engineering a Virus-Like Particle as an Antigenic Platform for a Pfs47-Targeted Malaria Transmission-Blocking Vaccine

**DOI:** 10.1038/s41598-019-53208-z

**Published:** 2019-11-14

**Authors:** Lampouguin Yenkoidiok-Douti, Adeline E. Williams, Gaspar E. Canepa, Alvaro Molina-Cruz, Carolina Barillas-Mury

**Affiliations:** 1 Laboratory of Malaria and Vector Research, NIAID/NIH, Rockville, MD USA; 2 Fischell Department of Bioengineering, University of Maryland, College Park, MD USA; 3 Present Address: Department of Microbiology, Immunology, and Pathology, Colorado State University, Fort Collins, CO USA

**Keywords:** Vaccines, Parasitology

## Abstract

We recently characterized Pfs47, a protein expressed on the surface of sexual stages and ookinetes of *Plasmodium falciparum*, as a malaria transmission-blocking vaccine (TBV) target. Mice immunization induced antibodies that conferred strong transmission-reducing activity (TRA) at a concentration of 200 μg/mL. Here, we sought to optimize the Pfs47 vaccine to elicit higher titers of high-affinity antibodies, capable of inducing strong TRA at a lower concentration. We report the development and evaluation of a Pfs47-based virus-like particle (VLP) vaccine generated by conjugating our 58 amino acid Pfs47 antigen to Acinetobacter phage AP205-VLP using the SpyCatcher:SpyTag adaptor system. AP205-Pfs47 complexes (VLP-P47) formed particles of ~22 nm diameter that reacted with polyclonal anti-Pfs47 antibodies, indicating that the antigen was accessible on the surface of the particle. Mice immunized with VLP-P47 followed by a boost with Pfs47 monomer induced significantly higher antibody titers, with higher binding affinity to Pfs47, than mice that received two immunizations with either VLP-P47 (VLP-P47/VLP-P47) or the Pfs47 monomer (P47/P47). Purified IgG from VLP-P47/P47 mice had strong TRA (83–98%) at concentrations as low as 5 μg/mL. These results indicate that conjugating the Pfs47 antigen to AP205-VLP significantly enhanced antigenicity and confirm the potential of Pfs47 as a TBV candidate.

## Introduction

Although malaria-related mortality has decreased over the last two decades, these gains have stalled in the past two years. According to the *World Malaria Report* of 2018, the number of malaria cases increased from 217 million cases in 2016 to 219 million cases in 2017, resulting in 435,000 deaths^1,2^. *Plasmodium falciparum* causes most human malaria morbidity and mortality and is transmitted by *Anopheles* mosquitoes. Current control efforts are threatened as mosquitoes and *Plasmodium* parasites exhibit resistance to insecticides and anti-malarial drugs, respectively^2^. Thus, reducing disease transmission through novel control strategies could be key to eliminating malaria.

The natural life cycle of *Plasmodium* parasites within the mosquito vector can be exploited to prevent malaria transmission. When a mosquito takes an infectious bloodmeal, it ingests thousands of sexual-stage gametocytes that egress from red blood cells, develop into mature gametes and undergo fertilization in the midgut lumen. As they become extracellular, gametes are vulnerable to effector molecules, such as antibodies or complement, present in the bloodmeal. Parasites suffer dramatic loses in the mosquito which results in population bottlenecks, making the mosquito stages of the parasite attractive targets to develop innovative strategies to disrupt malaria transmission^3,4^.

Most transmission-blocking vaccines (TBV) induce functional antibodies in the human host that target *Plasmodium* surface proteins essential for parasite development in the mosquito^5,6^. Two of the leading TBV targets, Pfs48/45 and Pfs230, as well as Pfs47, are members of the 6-cysteine family of proteins that are expressed on the surface of *Plasmodium* gametes. Antibodies against these proteins prevent *Plasmodium* fertilization and ookinete formation^7,8^.

We recently showed that Pfs47 is a promising transmission-blocking vaccine target^8^. Pfs47 mediates parasite evasion of the mosquito immune system, and its homologue in *Plasmodium berghei* has been shown to be required for female gamete fertility^3,8–10^. Pfs47 has three domains, and mice immunized with full length Pfs47 elicited a strong antibody response to domains 1 and 3. These antibodies, however, did not confer significant transmission-reducing activity (TRA), defined as the % inhibition in mean oocyst count per mosquito, in *Anopheles gambiae* infected with *P. falciparum*^8^. However, a series of deletions in the polypeptide and mapping with monoclonal antibodies revealed that antibodies that bind to a 58 amino acid region of Pfs47 in domain 2 did confer strong TRA (78–99%) at concentrations of 200–300 μg/mL. Because the protective Pfs47 antigen is small, we explored whether multimerization via linkage to a carrier could enhance its immunogenicity.

Virus-like particles (VLPs) are self-assembling, non-infectious, multimeric proteins that resemble the structural organization and conformation of viruses^11^. They are highly immunogenic due to their size and repetitive antigen display^11^. Their small size, ranging from 20–200 nm, enhances drainage to the draining lymph nodes - specialized tissues where immune cell activation occurs - and facilitates antigen uptake by antigen-presenting cells^11^. Their dense geometric structure induces strong Toll-like receptor (TLR) signaling and potent B-cell receptor clustering^12,13^. Finally, VLPs encapsulate nucleic acids, which can induce additional TLR signaling^13^.

VLPs can be engineered to display foreign surface antigens in their native conformation, and they have been shown to enhance immunogenicity^14^. There are several methods to link antigens to VLPs, including genetic fusion, chemical conjugation, and the recently developed “plug-and-display” AP205-SpyCatcher:SpyTag protein adaptor system^14,15^. The former two methods are challenging because direct linkage of a foreign protein to a VLP may disrupt particle expression and self-assembly^14^.

The versatile AP205-SpyCatcher:SpyTag conjugation system, however, offers several advantages^15^. AP205 is an RNA bacteriophage coat protein that can be genetically fused to a 15 kDa protein adaptor “SpyCatcher” without interfering with VLP self-assembly^15,16^. A small coupling partner peptide “SpyTag” can be genetically fused to a variety of antigenic proteins. AP205-SpyCatcher forms a covalent peptide bond with the SpyTag conjugated to the peptide in an *in vitro* reaction that occurs under a wide variety of conditions. Thus, this system allows for effective conjugation of the AP205 VLPs with foreign antigens and minimizes the pitfalls of traditional linkage methods.

In a recent study comparing the efficacy of three VLP platforms, the AP205-SpyCatcher:SpyTag system induced the highest quality functional antibodies against the TBV candidate Pfs25, a vaccine that targets the ookinete stage of *Plasmodium*^17^. Antibodies after immunization with AP205-SpyCatcher:SpyTag-Pfs25 had 95–100% TRA at concentrations as low as 30 μg/mL of total IgG, while antibodies obtained after immunization with Pfs25 monomer had much lower biological activity (51% TRA at 750 μg/mL)^17^. In this study, we aimed to improve the immunogenicity of the Pfs47 antigen – hereafter termed “P47”– via linkage with AP205-SpyCatcher VLPs. We hypothesized that dense P47 display on the surface of VLPs would enhance immunogenicity and generate a higher titer of high-affinity binding antibodies to native Pfs47 with stronger TRA.

## Results

### Protein expression, purification, and conjugation of AP205-SpyCatcher with SpyTag-P47 to form a fully antigen-coated virus-like particle

**Figure 1:**
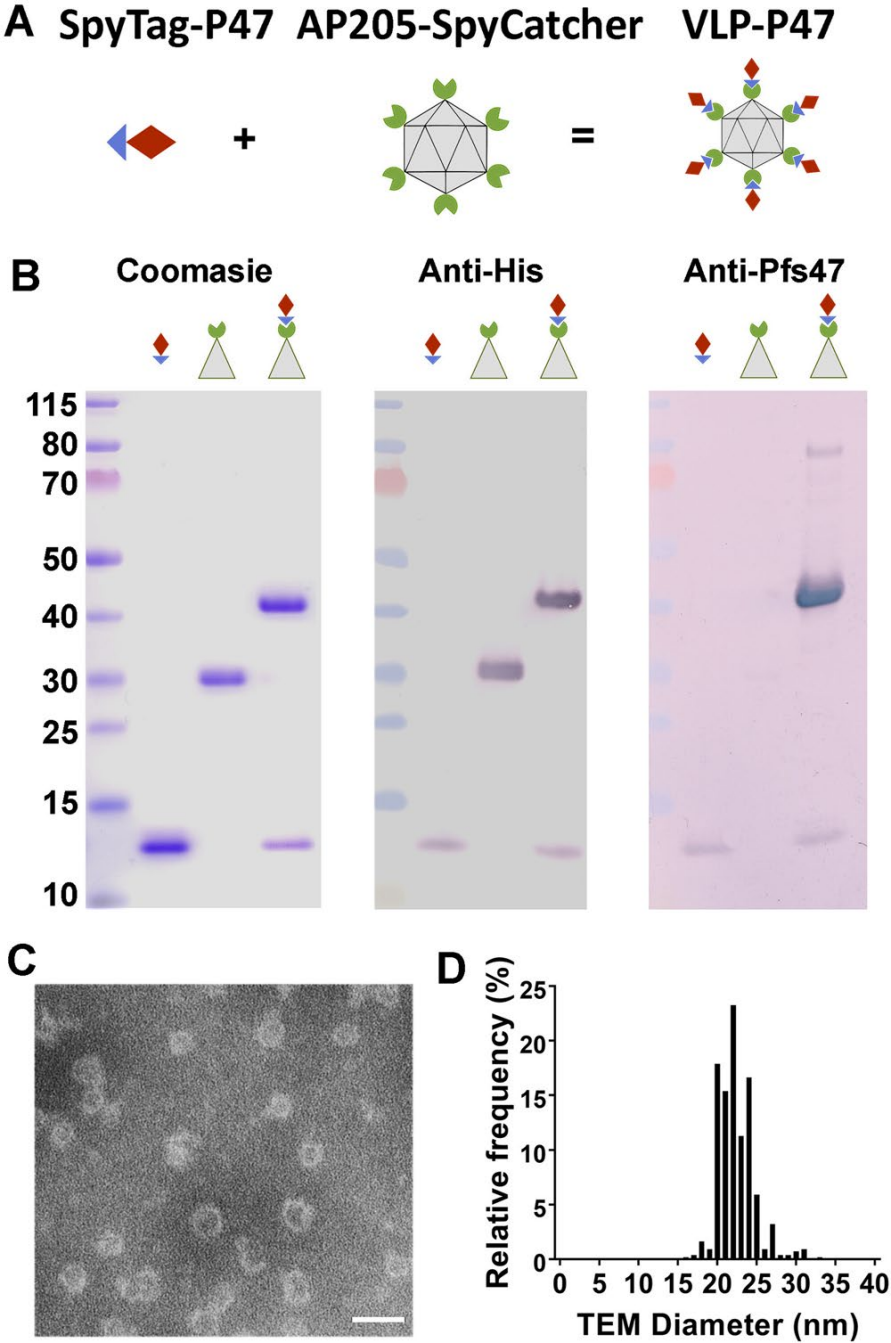
AP205-SpyCatcher and SpyTag-P47 isopeptide bond formation. (**A**) Schematic representation of the AP205-SpyCatcher and SpyTag-P47 isopeptide bond formation. Diagrams show SpyCatcher in green, Spytag in blue, and P47 in red. (**B**) Coomassie blue staining of SpyTag-P47, AP205-SpyCatcher, and conjugated VLP-P47 in SDS-PAGE after boiling and reducing in SDS-loading buffer (left). Anti-his western blot of SpyTag-P47, AP205-SpyCatcher, and conjugated P47-VLP (center). Anti-Pfs47 western blot of SpyTag-P47, AP205-SpyCatcher, and conjugated VLP-P47 (right). (**C**) TEM of VLP-P47 after negative staining with 2% uranyl acetate. (**D**) Size distribution of VLP-P47 from TEM image (n = 559). The average hydrodynamic diameter is 22.48 +/− 2.26 nm. Scale bar: 50 nm.

The P47 antigen was expressed as a recombinant protein and covalently linked to the surface of the AP205 VLP using the AP205-SpyCatcher:SpyTag platform (Fig. 1A)^15^. To generate AP205-SpyCatcher, a construct in which the ΔN1SpyCatcher was fused to the CP3 coat protein (His-tagged) of the AP205 RNA bacteriophage was designed (Fig. S1A), as previously described^15,16^. AP205-SpyCatcher was subcloned into both pET24 and pET17b, and both plasmids were used to transform both *E. coli* BL21 (DE3) pLysS (Thermofischer) and *E. coli* OverExpress™ C41(DE3) (Lucigen). AP205-SpyCatcher expression in *E. coli* BL21 (DE3) pLysS and *E. coli* OverExpress™ C41(DE3) was induced with 1 mM Isopropyl β-D-1 thiogalactopyranoside (IPTG) for 4 hours at 37 °C (Fig. S1B), as previously described^15^. AP205-SpyCatcher expression was monitored in soluble fractions and inclusion bodies of *E. coli* extracts in both cell expression systems by western blot analysis with anti-His antibody detection (Fig. S1C). We found that *E. coli* BL21 (DE3) pLysS cells transformed with pET17b-AP205-SpyCatcher had the highest expression level of soluble protein (Fig. S1C). To optimize protein yield, we harvested the cells at different times post-induction with IPTG and compared the yield at 37 °C and 30 °C. The best yield of soluble pET17b-AP205-SpyCatcher particle (~1 mg/L of culture) was obtained 6 h after inducing expression with 1 mM IPTG at 30 °C in BL21 (DE3) pLysS cells (Fig. S1D) and these conditions were used in all subsequent expressions.

We exploited the high molecular weight of AP205-VLP to remove irrelevant proteins in the *E. coli* extract by dialyzing it using a 300 kDa cutoff membrane before nickel affinity purification. Because multiple His-tags are present on the VLP surface (one for each of the ~180 monomers in each particle), we developed a protocol to purify the particle under high stringency conditions using high imidazole concentrations. Soluble AP205-SpyCatcher VLP was bound to a nickel affinity chromatography column in a buffer containing 50 mM imidazole and washed with 100 mM imidazole. Endotoxin was removed by including 0.1% Triton X-114 in the first washing step. This treatment was very effective and reduced endotoxin in purified recombinant proteins from >200 EU/ml to 0.35 EU/ml. The final purified protein was eluted with 2M imidazole, dialyzed in PBS pH 7.5, and the purity of the particle was confirmed by SDS-PAGE under denaturing and reducing conditions (Fig. S1E). The presence of a high molecular weight AP205-SpyCatcher VLP that contained both protein and nucleic acids was confirmed by native agarose gel electrophoresis (Fig. S2A)^15,18^. Nucleic acids are present because AP205 VLPs enclose host RNA as they fold. Negative-staining transmission electron microscopy (TEM) of AP205-SpyCatcher confirmed the presence of particles consistent with the size and morphology of VLPs (Fig. S2B)^18^.

A P47 recombinant protein containing the SpyTag peptide bound to the N-terminus (Fig. S3A) with a 3× (GSG) linker sequence was also expressed in *E. coli* BL21 (DE3) pLysS and purified using our previously described protocol^8^. SpyTag-P47 protein was also purified using nickel affinity chromatography, resulting in a single band of the expected size (~12 kDa) on an SDS-PAGE gel stained with Coomassie blue (Fig. S3B,C). Western blot analysis using monoclonal antibodies against Pfs47 confirmed that we successfully expressed soluble SpyTag-P47 protein (Fig. S3C).

Although AP205-SpyCatcher was stable in PBS pH 7.5, the particle was unstable and precipitated when conjugated with SpyTag-P47 at this pH. To solve this problem, we analyzed the stability of SpyTag-P47 at three different pHs (4.5, 6.5, and 8.5) in the presence or absence of 150 mM NaCl (Table S1). We found that the stability of SpyTag-P47 is pH dependent, with maximum stability (three weeks at 4 °C at a concentration of 200 μg/mL) at pH 4.5 with or without 150 mM NaCl. The linkage reaction was thus carried out in PBS buffer pH 4.5 without NaCl.

The optimal ratio to covalently link the antigen and saturate the particle surface was established by mixing different molar rations of SpyTag-P47 and AP205-SpyCatcher (between 3.2:1 to 0.9:1 molar ratio of SpyTag-P47: AP205-SpyCatcher). The linkage reaction was very effective, and at 1:1 molar ratio more than 95% of the AP205-SpyCatcher monomers reacted with the SpyTag-P47 antigen (Fig. S4). The AP205-SpyCatcher:SpyTag-P47 VLP particle was stable after conjugation and was stored in PBS pH 4.5 without NaCl.

The covalently bound AP205-SpyCatcher:SpyTag-P47 protein had the expected size (45 kDa) under denaturing and reducing conditions (Fig. 1B). Western blot analysis showed that the conjugated VLP strongly reacted with anti-Pfs47 monoclonal antibodies (Fig. 1B), while, as expected, no signal was observed for the unconjugated SpyCatcher-AP205 VLP (Fig. 1B). The SpyCatcher-AP205 protein is recognized by anti-His antibodies, and a band corresponding to the size of the unconjugated protein was no longer detected when the reaction was completed, indicating that the particle was decorated with P47 antigen (Fig. 1B). Conjugated AP205-SpyCatcher:SpyTag-P47 VLPs are henceforth referred to as “VLP-P47.” AP205 VLPs are T = 3 icosahedral particles with a diameter of ~20–30 nm^18^. Negative-staining transmission electron microscopy (TEM) of VLP-P47 confirmed that spherical particles with an average diameter of 22.48 +/− 2.26 nm, characteristic of AP205 VLPs (Fig. 1C,D), were present.

### VLP-P47 is more immunogenic than unconjugated P47 monomers

The immunogenicity of VLP-P47 was determined by vaccinating BALB/c mice with 1 µg equivalent P47 antigen either as monomer or as a VLP-P47. Three different immunization strategies were tested. Each group of mice (n = 5) was independently immunized with (1) P47 monomer and boosted with the same antigen (P47/P47), (2) VLP-P47 and boosted with the same antigen (VLP-P47/VLP-P47), or (3) VLP-P47 followed by a boost with P47 (VLP-P47/P47). Two weeks after the boost immunization, mice were sacrificed, and sera were collected to determine antibody responses.

**Figure 2:**
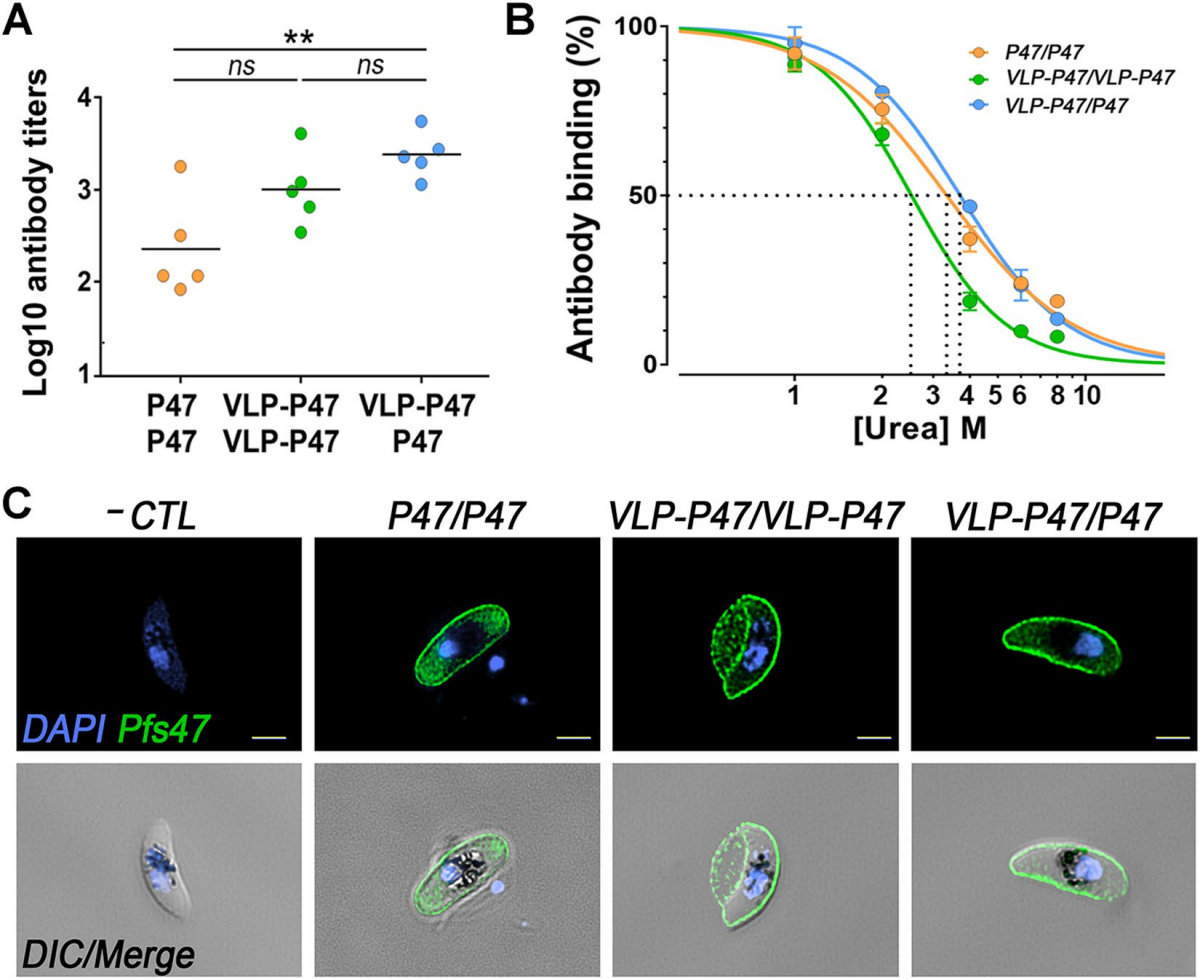
Immunogenicity of VLP-P47. (**A**) Levels of anti-Pfs47 specific antibodies measured in sera of individual mice (n = 5) immunized and boosted with 1 µg equivalent P47 per immunization. Serum was collected 14 days post-boost and titers were measured by ELISA. Mice (circles) and medians (bars) are shown. (**B**) Avidities of purified antibody responses were assessed by urea-displacement ELISA and is reported as the reduction in OD by incubation with increasing concentrations of urea (1–8 M) compared to incubation without urea. EC50 (concentration of urea required to reduce the OD405 to 50% of that without urea) was then determined using a sigmoidal dose response (non-linear) regression. *P ≤ 0.05, **P ≤ 0.01. (**C**) Immunofluorescence assay of *in vitro* cultured *P. falciparum* NF54 gametocytes stage IV stained with purified antibody obtained from the three immunization groups. As a negative control, gametocytes were stained with antibodies from naïve mice (top row). The staining was imaged using a confocal microscope; DNA was stained with DAPI (blue, left); Antibody binding was detected by Alexa Fluor 488-conjugated goat anti-mouse IgG (green, center); and a merge of DAPI and antibody staining with DIC view are also shown (right). Scale bar: 2 µm.

All immunization groups had a positive antibody response against Pfs47 in ELISA (Fig. 2A). The median antibody response of the groups immunized with VLP-P47/P47 was significantly higher than that of the group that was only immunized with the P47/P47 monomer (p = 0.0052) (Fig. 2A). There was no significant difference in antibody titers between mice that were immunized with VLP-P47 and were boosted with either VLP-P47 or P47 (Fig. 2A). Mouse serum derived from each immunization group was pooled together and IgG was purified. The reactivity of the purified IgG was also tested by ELISA. Mice that received at least one dose of VLP-47 (VLP-P47/VLP-P47 or VLP-P47/P47) had significantly higher anti-Pfs47 antibody titers than those that only received unconjugated P47 (p < 0.005) (Fig. S5). To assess the antibody reactivity against the P47 antigen and the VLP carrier after immunization with the VLP-P47 particle, we performed serial dilution ELISAs with purified IgGs from mice immunized with VLP-P47/VLP-P47 or VLP-P47/P47, coating plates with the same molar ratio of either P47 monomer or unconjugated VLP. Mice immunized with at least one dose of VLP-P47 have strong ELISA reactivity to the VLP carrier that is one order of magnitude stronger than the reactivity to the P47 monomer (Fig. S6).

The avidity of the purified IgG was measured using a chaotropic agent (urea) to disrupt the interactions between the antibody and the coating antigen in ELISA assays. ELISA optical densities (OD) with and without increasing concentrations of urea were compared. If an antibody binds weakly to an antigen, the binding will be disrupted by a lower urea concentration. The avidity EC50, defined as the amount of urea required to decrease the OD by 50% (Fig. 2B), was determined for each IgG pool^17^. Interestingly, antibodies obtained after VLP-P47/P47 immunization had significantly higher avidity (EC50 = 3.71 M) than VLP-P47/VLP-P47 antibodies (EC50 = 2.51 M) (p < 0.01) but did not significantly differ from P47/P47 antibodies (EC50 = 3.31 M) (p = 0.07) (Fig. 2B).

### Vaccine-induced antibodies recognize native Pfs47 antigen

Pfs47 is expressed on the surface of male and female gametocytes and ookinetes present in the midgut of the mosquito vector^8,9^. We assessed the ability of polyclonal antibodies against the recombinant Pfs47 antigen to recognize native Pfs47 on the surface of parasites via immunofluorescence assays (Fig. 2C). Purified antibodies from each vaccine group recognized Pfs47 on the surface of gametocytes. Antibodies purified from naïve mice were used as a negative control.

### Mice immunized with VLP-P47 and boosted with unconjugated P47 monomer elicit antibodies with strong transmission-reducing activity (TRA)

**Figure 3:**
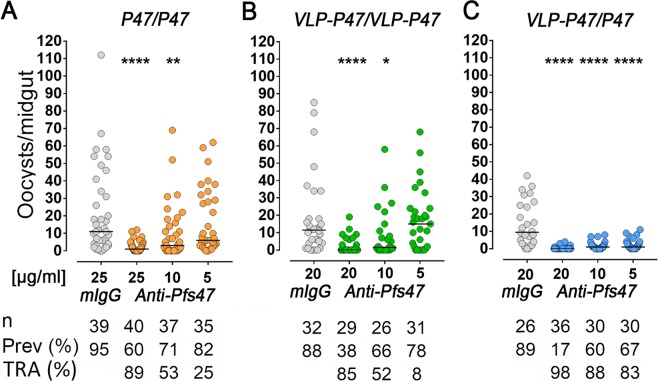
Antibodies generated from immunization with VLP-P47 and boosted with P47 monomer elicit antibodies with enhanced TRA at low concentrations as compared to antibodies against P47 alone. Transmission reducing activity of purified mouse polyclonal IgG at decreasing concentrations against Pfs47 obtained after immunization with the three immunization groups tested by standard membrane feeding assay (SMFA). Dots represent the number of oocysts in individual mosquitoes and the lines indicate median infection. Number of mosquitoes dissected (n); infection prevalence (prevalence); transmission reducing activity (TRA) as percent inhibition of infection intensity in an SMFA relative to IgG control purified from naïve mice (mIgG). Medians were compared using the Mann–Whitney test: *P < 0.05; **P < 0.01; ****P < 0.0001).

The TRA of IgG from the three immunization groups (P47/P47, VLP-P47/VLP-P47, or VLP-P47/P47) was assessed by standard membrane feeding assays (SMFA). Total IgG purified from pooled sera was mixed with *P. falciparum* gametocyte cultures at different final concentrations (25 or 20 μg/mL, 10 μg/mL and 5 μg/mL) and fed to *A. gambiae* mosquitoes. IgG from mice immunized with P47/P47 or VLP-P47/VLP-P47 had strong TRA, 89% at 25 μg/mL and 85% at 20 μg/mL, respectively. However, the TRAs dropped at lower IgG concentrations (10 μg/mL and 5 μg/mL IgG) to 53% and 25% for the P47/P47 group and to 52% and 8% for the VLP-P47/VLP-P47 group (Fig. 3). Furthermore, in both replicates for the P47/P47 and VLP-P47/VLP-P47 groups (Figs 3A,B and S7A,B), the median number of parasites with IgG at 20–25 μg/mL and 10 μg/mL were significantly lower than the controls, while at 5 μg/mL the differences were no longer significant. IgG from mice immunized with VLP-P47/P47 exhibited the strongest TRA with 98%, 88% and 83% TRA at 20 μg/mL, 10 μg/mL and 5 μg/mL IgG, respectively, in assays when the infection level in the control groups that received IgG from naïve mice was ~10 oocysts/midgut (Fig. 3C). As expected, when the infection level in the control group was higher (~20 oocysts/midgut) the TRA was slightly lower, but the VLP-P47/P47 IgG still exhibited strong TRA, with 95%, 83% and 60% TRA at 20 μg/mL, 10 μg/mL and 5 μg/mL IgG, respectively (Fig. S7). For both experiments with the VLP-P47/P47 group (Figs 3C and S7C) the median level of infection was still significantly lower than the control with IgG as low as at 5 μg/mL.

## Discussion

We have previously identified a target region of Pfs47 where antibody binding disrupts *A. gambiae* infection with *P. falciparum* – termed “P47” in this manuscript– and have shown that it is a promising malaria transmission-blocking vaccine target^8^. In this study, we aimed to enhance P47 immunogenicity. Efforts to enhance immune responses to other malaria TBVs have involved multimerization of the antigen on nanoparticles^15,17,19–21^. Here, we conjugated our 58 amino acid P47 antigen on the surface of AP205 virus-like particles. We chose VLPs due to their ability to display native proteins on their surfaces, and the efficiency with which they are endocytosed and cross-presented to T cells in the context of major histocompatibility complex classes I and II^11,15^. We demonstrate that conjugating P47 to a AP205 VLP using the SpyCatcher:SpyTag system results in correctly folded particles that are saturated with P47 antigen (Fig. 1).

VLP-P47 elicited a robust antibody response against Pfs47 in mice that received a booster dose with either VLP-P47 (VLP-P47/VLP-P47) or P47 alone (VLP-P47/P47) (Figs 2 and S5). However, only the VLP-P47/P47 group displayed significantly higher antibody titers than mice immunized with unconjugated monomeric P47 (Fig. 2A,B). This indicates that, as expected, P47 was more immunogenic when it was displayed on the VLP surface. Although VLP-P47/P47 elicited antibodies with significantly higher titers than P47/P47, there was no significant difference between their binding avidities (Fig. 2C). This is different from a previous report where immunization with AP205-Pfs25 elicited significantly higher antibody titers, and the antibodies also had stronger avidity than those derived after immunization with monomeric Pfs25^17^.

Although P47/P47 immunization elicited lower antibody titers, receiving a booster dose with P47 monomer (P47/P47 of VLP-P47/P47) significantly enhanced the avidity of the antibodies to P47. This observation suggests that although the P47 monomer is less immunogenic, it presents epitopes that elicit antibodies with stronger binding to the antigen. Although the IgG concentration is adjusted to compare different polyclonal antibodies in the SMFA, the TRA depends, in part, on the proportion of the antibodies that are directed to P47. Indeed, immunization with the VLP-P47 particle elicited ELISA reactivity to the VLP carrier that was 10-fold higher than to the P47 antigen. Because our antigen is small (58 aa), the exposed area of the VLP may be much larger than that of the P47 peptide decorating the particle, and this may enhance the antibody response to the carrier. The VLP-P47/P47 immunization had the strongest TRA, probably because the antigen conjugated to the VLP induced a strong initial antibody response, while the booster dose with P47 monomer focused the antibody response against P47. For small antigens, a smaller carrier may be more effective eliciting a strong immune response to the protective antigen rather than to the immunization platform. Together, the findings presented in this manuscript provide further evidence for the potential of Pfs47 as a transmission blocking vaccine target.

## Methods

### Cloning

SpyCatcher-AP205 was synthesized by GenScript and was cloned into pET17b and pET24 vectors with the following order: ΔN1SpyCatcher^15,16^, (GSG)_3_ linker, codon-optimized AP205 coat protein 3^15^, and C-terminal His_6_ (Fig. S1A). SpyTag-P47 was cloned into pET17b with the following order: SpyTag^15^, (GSG)_3_ linker, P47^8^, and C-terminal His_6_ (Fig. S2A)_._ Cloning was performed with In-Fusion HD kits (Clontech) and inserts were confirmed by Sanger sequencing.

### Expression of AP205-SpyCatcher

SpyCatcher-AP205 was expressed by either Brune *et al*.^15^ or by an adapted protocol. Briefly, BL21(DE3) pLysS chemically competent *E. coli* cells (ThermoFisher) or *E. coli* OverExpress™ C41(DE3) (Lucigen) were transformed with pEt17b-AP205-SpyCatcher or pET24-AP205-SpyCatcher and inoculated onto LB agar plates with 100 µg/mL ampicillin and 34 µg/mL chloramphenicol. The plates were incubated O/N at 37 °C. Ten transformed BL21 colonies were picked and cultured in 10 separate tubes containing 4 mL LB supplemented with 100 µg/mL ampicillin and 34 µg/mL chloramphenicol O/N at 37 °C. These cultures were added to 1 L of LB broth supplemented with 100 µg/mL ampicillin and 34 µg/mL chloramphenicol and shook at 160 RPM at 37 °C until A600 was 0.6–0.9. Expression was induced with 1 mM IPTG for 6 h at 30 °C. The culture was then centrifuged at 4000 × g for 20 m at 4 °C. The resulting pellet was resuspended with 50 mL cold PBS, spun again for 10 m, and left frozen at −20 °C O/N. Expression was tested by anti-His_6_ western blot in both the soluble fractions (SF) and inclusion bodies (IB).

### Purification of AP205-SpyCatcher

Soluble AP205-SpyCatcher was purified following an adapted protocol based on Brune *et al*.^15^. The 1 L pellets were resuspended at RT in 30 mL hepes-buffered saline (HBS) lysis buffer (20 mM Hepes, 150 mM NaCl, 0.1% triton-X-100, 0.1% Tween 20) and incubated on ice for 10–15 m. The pellets were sonicated 4X for 30 s–2 m. The lysates were then spun twice at 15,000 × g for 20 m at 4 °C. The supernatants were then filtered through 0.45 µm SFCA filters (ThermoFisher) and dialyzed O/N in the same solution using a 300 kDa cut-off membrane. To bring lysates to 50 mM imidazole, appropriate volumes of Ni-NTA elution buffer (20 mM Hepes, 150 mM NaCl, and 1 M imidazole) were added to the filtered lysates, along with 500U benzonase (Sigma Aldrich) and 3 mL HisPur Ni-NTA resin (Life Technologies). The lysates were left slowly rocking at 4 °C for 20–25 m, added to two 12 mL chromatography columns (BioRad), and filtered by gravity. The resin was washed with 40–50 mLs 100 mM imidazole buffer (20 mM Hepes, 150 mM NaCl, 100 mM imidazole), and the proteins eluted into 500 µL fractions with 2 M imidazole, 20 mM Hepes, 150 mM NaCl, and 0.1% Tween 20. Before elution, the lysate was washed with 50 mL washing buffer containing 20 mM Hepes, 150 mM NaCl, 100 mM imidazole and 0.1% (v/v) Triton X-114 (TX114), to remove endotoxin^21,22^. Elutes were dialyzed with a 300 kDa MWCO Spectrum™ Spectra/Por™ Float-A-Lyzer™ G2 Dialysis Device (ThermoFisher) in 20 mM Hepes, 150 mM NaCl, and 0.1% Tween 20. The AP205-SpyCatcher protein underwent a final dialysis with the same 300 kDa MWCO dialysis device (ThermoFisher) into PBS pH 4.5 to equilibrate the buffer with that of the SpyTag-P47 protein.

### Expression of SpyTag-P47

BL21(DE3) pLysS chemically competent *E. coli* cells were transformed with pet17b-SpyTag-P47. One colony was cultured in 4 mL media O/N at 37 °C and was added to 100 mL as described above. Expression was induced with 1 mM IPTG for 4 h at 37 °C at A600 ~ 0.4–0.6. The culture was then centrifuged at 4000 × g for 20 m at 4 °C and the pellet was left frozen at −20 °C O/N.

### Purification of SpyTag-P47

The SpyTag-P47 pellet was resuspended in 20 mL PBS and sonicated 4X for 30 s–2 m. Lysates were then centrifuged at 35000 × g for 15 m at 4 °C. Resulting pellets were resuspended in PBS and 1% triton and sonicated 4X for 30 s–2 m. The suspension was again centrifuged at 35000 × g for 15 m at 4 °C. The resulting inclusion body pellet was resuspended in PBS 6 M Urea, 1 M NaCl, and 20 mM imidazole and left on ice for 30m-2h. The suspension was then pelleted at 35000 × g for 15 m at 4 °C.

The supernatant was added to 1 mL HisPur Ni-NTA resin (Life Technologies) that had been equilibrated in a 12 mL chromatography column (BioRad). The resin was washed with 25 mL equilibration buffer (PBS 6 M Urea, 1 M NaCl, 20 mM imidazole) and subsequently washed with 10 mL washing buffer (PBS 2 M Urea, 1 M NaCl, 20 mM imidazole). The protein was eluted into 1 mL fractions with PBS 2 M Urea, 1 M NaCl, 300 mM imidazole. Elutes were passed through a PD10 desalting column at pH 4.5.

### SpyCatcher-AP205:SpyTag-P47 conjugation

Reactions occurred at RT for 3 h in a total volume of 25 µL. Different molar ratios of SpyTag-P47/AP205-SpyCatcher were tested, and PBS pH 4.5 was used to bring reaction volume to 25 µL. The reaction was stopped by adding 1X LDS sample buffer and NuPAGE Sample Reducing Agent (ThermoFisher) and was analyzed by SDS-PAGE.

### SDS-PAGE

All proteins were boiled under reducing conditions in 1X LDS (ThermoFisher) and were separated using 4–12% Bis-Tris protein gels (ThermoFisher). Gels were stained with Coomassie Blue. Protein concentrations were determined using Bradford protein assay (ThermoFisher).

### Western blot

Protein gels were transferred onto nitrocellulose membranes, which were blocked in 5% powdered milk (Sigma-Aldrich), 50 mM Tris-Cl, 150 mM NaCl, 1% Tween 20 (TBST) at RT. Membranes incubated O/N at 4 °C with 6× -HisTag Monoclonal Antibody (ThermoFisher, 1:5000) or with a monoclonal mouse anti-P47-IB2 (3 mg/mL, diluted 1:1000)^8^. Membranes were washed with TBST and incubated at RT for 2 h with goat anti-mouse antibody conjugated to alkaline phosphatase (1 mg/mL, diluted 1:5000). Membranes were washed again and proteins were detected for 5 m after using Western Blue Stabilized alkaline phosphatase substrate (Promega).

### Native-agarose gel electrophoresis

SpyCatcher-AP205 and VLP-P47 were run under native conditions in an agarose gel following Brune *et al*.^15^. Briefly, a 1% agarose gel prepared in 40 mM Tris, 20 mM acetic acid, 1 mM EDTA, pH 8 containing 1x SybrSafe (Life Technologies) was loaded with 40 µg of VLP and ran at 120 V. The gel was imaged with UV light and then stained in SimplyBlue (Invitrogen) to visualize protein.

### Transmission electron microscopy (TEM)

AP205-SpyCatcher or VLP-P47 (10 µL at a concentration of 0.2 mg/mL) were applied to 200 mesh copper grids with a thin carbon film (Electron Microscopy Sciences) and were glow discharged for 1 m before negative staining with 2% aqueous uranyl acetate followed by a brief water rinse. Grids were imaged at 80 kV with Hitachi HT-7800 camera and an XR81-B CCD detector (AMT) set at a pixel size of 0.52 nm (nominal magnification of 30,000 X). Particle size was modeled with IMOD^23^ and size distribution plot was made using GraphPad Prism software.

### Animals

All animal procedures were performed according to protocols approved by the NIAID and NIH Animal Care and Use Committee. 5–8 weeks old naïve, female BALB/c mice were purchased from Charles River (Germantown, MD) and maintained at a facility at the NIH.

Public Health Service Animal Welfare Assurance #A4149-01 guidelines were followed according to the National Institutes of Health Animal (NIH) Office of Animal Care and Use (OACU). These studies were done according to the NIH animal study protocol (ASP) approved by the NIH Animal Care and User Committee (ACUC), with approval ID ASP-LMVR5.

### Mice immunizations

Groups (n = 5) of female BALB/c mice were immunized intramuscularly in the rear thigh with a 50 µL vaccine containing a total of 1 μg of P47 antigen, either as monomer or conjugated as a VLP-P47 particle, both formulated with Magic Mouse adjuvant (Creative Diagnostics # CDN-A001). The immunized mice were boosted 4 weeks after the primary injection with the same dose of vaccine in alternative limbs. Endotoxin levels were quantified using Pierce LAL Chromogenic Endotoxin Quantitation Kit (Thermo Fisher Scientific) and were below 1 Unit/mL.

### Mosquitoes and parasites

*Anopheles gambiae* G3 were reared at 27 °C and 80% humidity on a 12 h light-dark cycle under standard laboratory conditions. The *Plasmodium falciparum* strain NF54 was maintained in O+ human erythrocytes using RPMI 1640 medium supplemented with 25 mM HEPES, 50 mg/l hypoxanthine, 25 mM NaHCO3, and 10% (v/v) heat-inactivated type O+ human serum (Interstate Blood Bank, Inc., Memphis, TN) at 37 °C with a gas mixture of 5% O2, 5% CO2, and balance N210.

### Standard membrane feeding assay

Mosquitoes were artificially infected with *P. falciparum* gametocyte cultures by membrane feeding as previously described^10^. *P. falciparum* NF54 cultures containing stage V gametocytes were used as the source of infective parasites. Gametocyte cultures were diluted with O+ human RBC and heat-inactivated O+ serum to achieve a 0.15–0.2% stage V gametocyte concentration and 42% hematocrit in 244 μL. Sera from each experimental group were pooled and antibodies were purified to create test samples. 16 µl purified IgG from test samples or naïve controls were diluted in AB+ human sera and used to feed mosquitoes using membrane feeders at 37 °C for 30 m. Eight days later, mercurochrome-stained oocysts were counted on midguts dissected from mosquitoes. Transmission reducing activity was estimated as the percent decrease in mean oocyst load in test groups compared to the naïve control group. Naïve mouse and human sera were purchased from Sigma-Aldrich (St. Louis, MO, USA) and Interstate Blood Bank (Memphis, USA), respectively.

### Statistical analysis

Statistical analyses were performed using Prism 6 (GraphPad). Differences between two groups were compared using a Mann-Whitney U test for non-normal distributions. One-way analysis of variance with Tukey post-test was used to compare differences between more than two groups.

## Supplementary information

Supplementary Information
