# Supplementary material for: Engineering a Virus-Like Particle as an Antigenic Platform for a Pfs47-Targeted Malaria Transmission-Blocking Vaccine
Source: Sci Rep. 2019 Nov 14;9:16833. doi: 10.1038/s41598-019-53208-z (PMC6856133; doi:10.1038/s41598-019-53208-z)
Supplement: Supplementary file 1 — Supplementary Information [file 41598_2019_53208_MOESM1_ESM.pdf]

Supplementary Information

Engineering Virus-Like Particles as an Antigenic Platform for a Pfs47-Targeted Malaria Transmission-Blocking Vaccine

Lampouguin Yenkoidiok-Douti,[1,2,*] Adeline E. Williams,[1,3,*] Gaspar E. Canepa,[1] Alvaro Molina-Cruz,[1] and Carolina Barillas-Mury[1,#]

[1]Laboratory of Malaria and Vector Research, NIAID/NIH, Twinbrook, MD, USA.

[2]Fischell Department of Bioengineering, University of Maryland, College Park, MD, USA.

[3]Department of microbiology, immunology, and pathology, Colorado State University, Fort Collins, CO, USA.

[#]Correspondence and requests for materials should be addressed to C. B-M. (email: cbarillas@niaid.nih.gov)

[*]These authors contributed equally to this work

Supplementary Figures

- Figure S1
- Figure S2
- Figure S3
- Figure S4
- Figure S5
- Figure S6
- Table S1

Supplementary Methods

- ELISAs
- IFA

A

>AP205-SpyCatcher Sequence

M*DSATHIKFSKRDEDGKELAGATMELRDSSGKTISTWISDGQVKDFYLYPGKYTFVETAAPDGYEVATAITFT*
*VNEQGQVTVNGKATKGDAHI*GSGGSGGSGANKPMQPITSTANKIVWSDPTRLSTTFSASLLRQRVKVGIAE
LNNVSGQYVSVYKRPAPKPEGCADACVIMPNENQSIRTVISGSAENLATLKAEWETHKRNVDTLFASGNAGL
GFLDPTAAIVSSDTTAHHHHHH

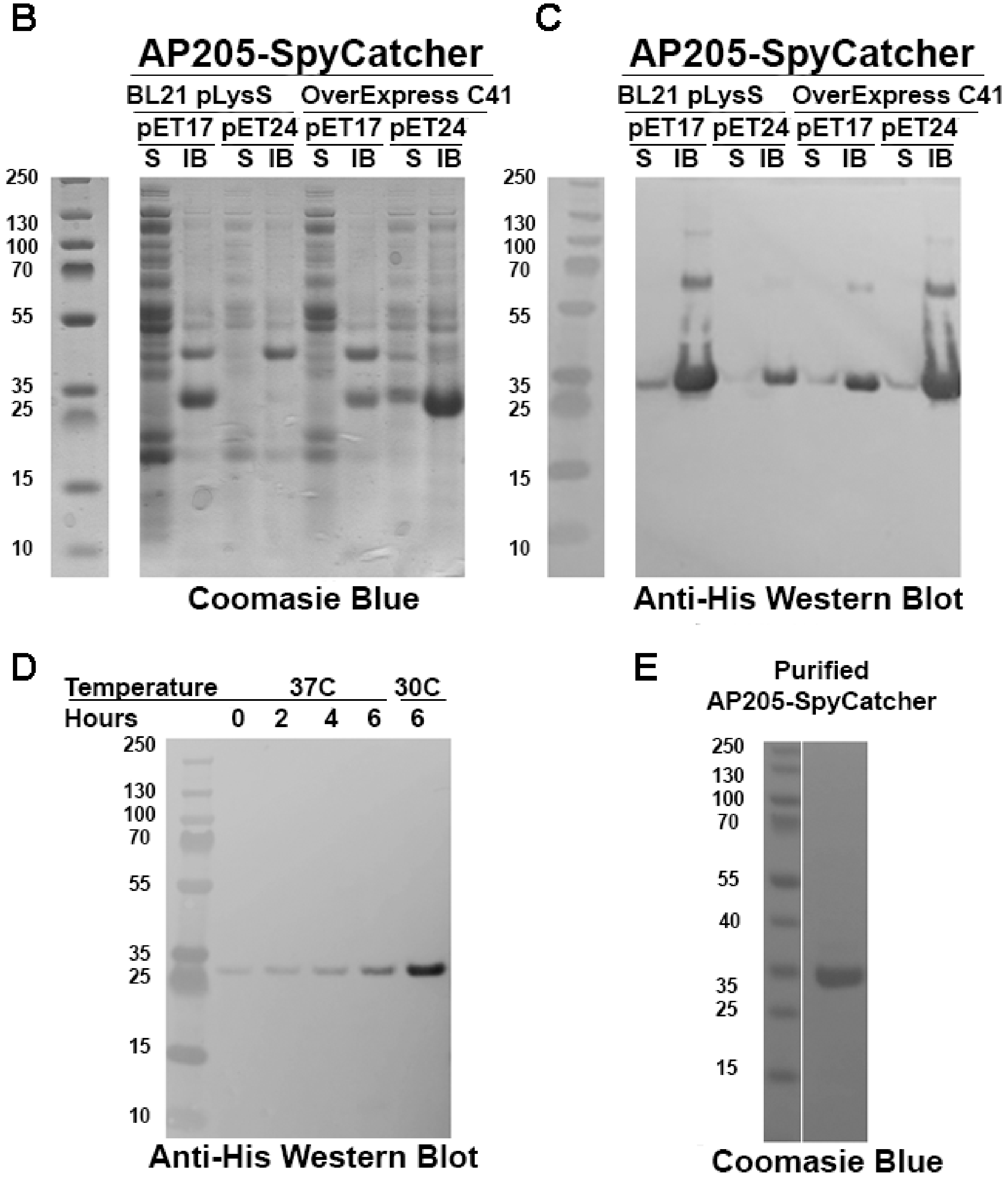

Figure S1. Sequence, expression, and purification of AP205-SpyCatcher.
(A) Protein sequence of AP205-SpyCatcher (amino acid residues in italics correspond to SpyCatcher and underlined amino acid residues to AP205). (B) Soluble fraction (S) and inclusion body (IB) preparation on a Coomassie blue-stained reducing SDS-PAGE gel illustrating the protein pattern using different combinations of either pET17- AP205-SpyCatcher or pET24- AP205-SpyCatcher in *E. coli* BL21 (DE3) pLysS (Thermofischer) and *E. coli* OverExpress™ C41(DE3) (Lucigen) independently after 4h induction with 1mM IPTG. (C) Soluble fraction (S) and inclusion body (IB) preparation on an anti-His Western Blot gel illustrating protein patterns using different combinations of either pET17-AP205-SpyCatcher or pET24- AP205-SpyCatcher in *E. coli* BL21 (DE3) pLysS (Thermofischer) and *E. coli* OverExpress™ C41(DE3) (Lucigen) independently after 4h induction with 1 mM IPTG. The unlabeled lanes show molecular weight standards. (D) AP205-SpyCatcher 1mM IPTG induction temperature time-course assay anti-His6 Western Blot showing the reactivity of pET17b-SpyCatcher-AP205 in *E. coli* BL21 (DE3) pLysS. (E) Coomassie blue-stained reducing SDS-PAGE gel illustrating dialyzed purified AP205-SpyCatcher.

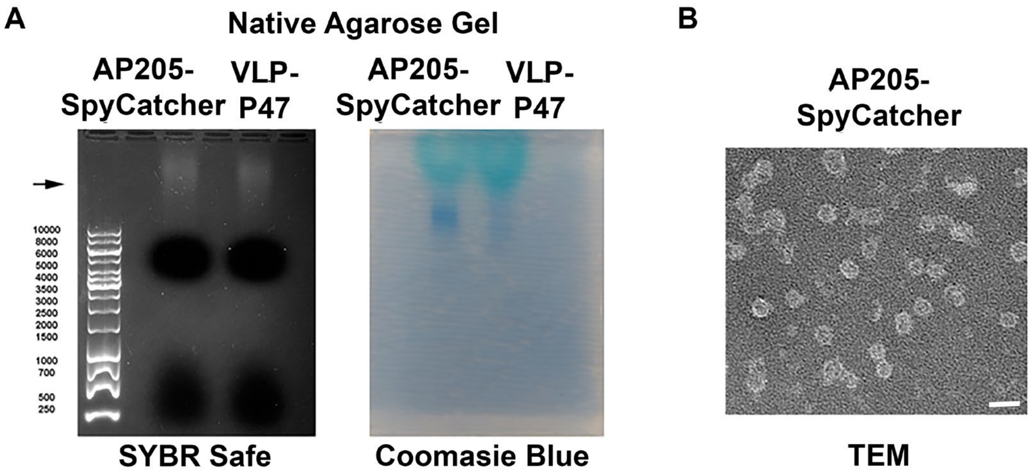

Figure S2. Analyzing of AP205-SpyCatcher and VLP-P47 expression.

(A) Native agarose gel of AP205-SpyCatcher and VLP-P47. The gel was stained with SybrSafe to stain for nucleic acid (right) or with a Coomassie Blue-based stain (SimplyBlue) to stain for protein (left). (B) TEM of AP205-SpyCatcher after negative staining with 2% uranyl acetate. Scale bar 20 nm

A

> SpyTag-P47 Sequence
M*AHIVMVDAYKPTK*GSGGSGGSGILRNQYNNIIELEKTKHIIHNKKDTYKYDIKLKESDILMFYMKEETIVESG
NAEEILNHHHHHH

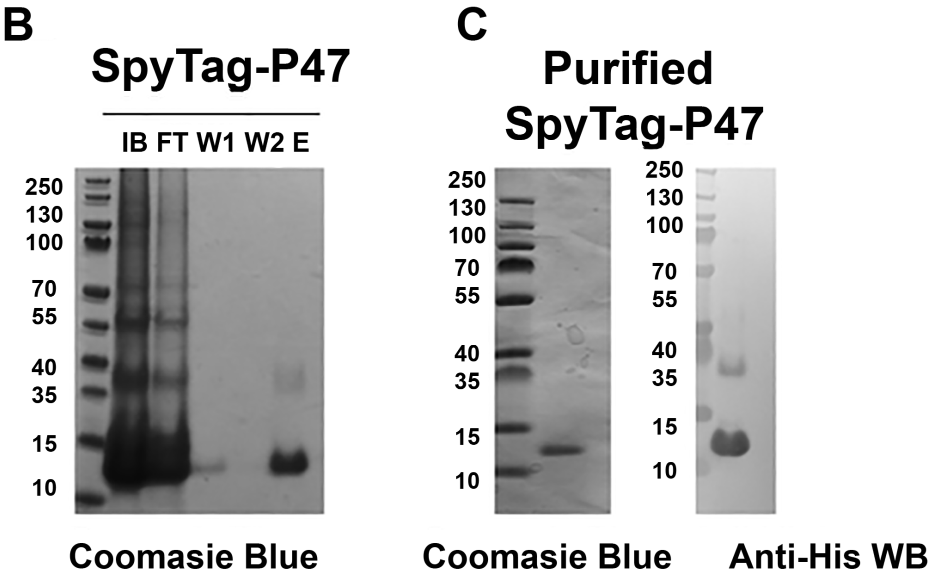

Figure S3. Sequence, expression and purification of SpyTag-P47.

(A) Protein sequence of SpyTag-P47 (amino acid residues in italics correspond to SpyTag and underlined amino acid residues to *P. falciparum* Pfs47 (Ile178-Asn235). (B) Steps of the expression and purification of SpyTag-1G. Lane 1, induced cells inclusion bodies; lane 2, flow through, lane 3-4 wash; lane 5, 1ml fraction of 300 mM imidazole eluted SpyTag-P47. The unlabeled lanes show molecular weight standards. (C) Coomassie blue-stained reducing SDS-PAGE gel illustrating dialyzed purified SpyTag-1G (right) and anti-Pfs47 Western Blot showing the reactivity of purified SpyTag-P47 (left).

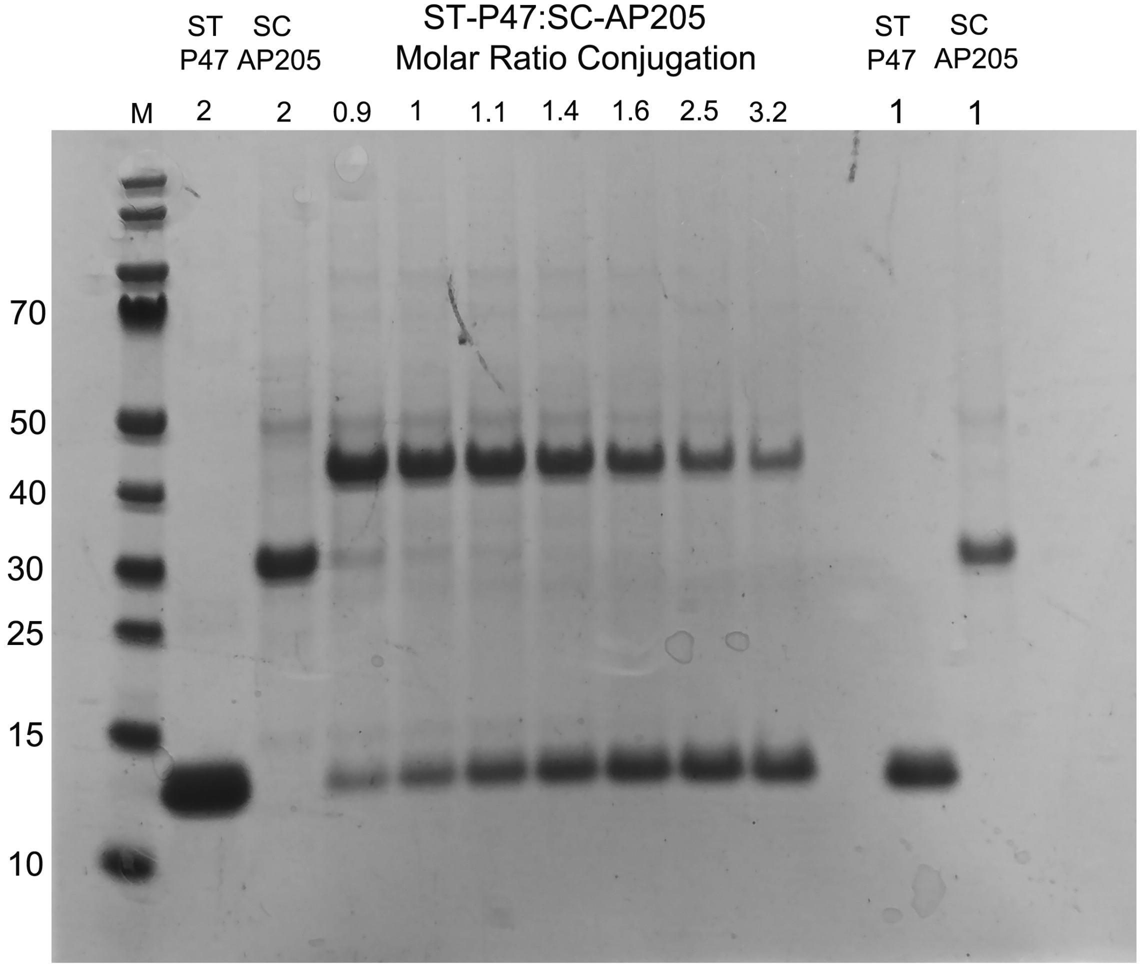

Figure S4. Reaction of AP205-SpyCatcher with SpyTag-P47.

Coomassie Blue-stained SDS-PAGE under reducing conditions of AP205-SpyCatcher and SpyTag-P47 reactions at different molar ratios incubated for 3 hours at 30°C. M, protein ladder; ST-P47, SpyTag-P47; SC-AP205, AP205-SpyCatcher; 0.9, 1.0, 1.1, 1.4, 1.6, 2.5 and 3.2 corresponds to the molar ratio used in the reaction between SpyTag-P47 and AP205-SpyCatcher.

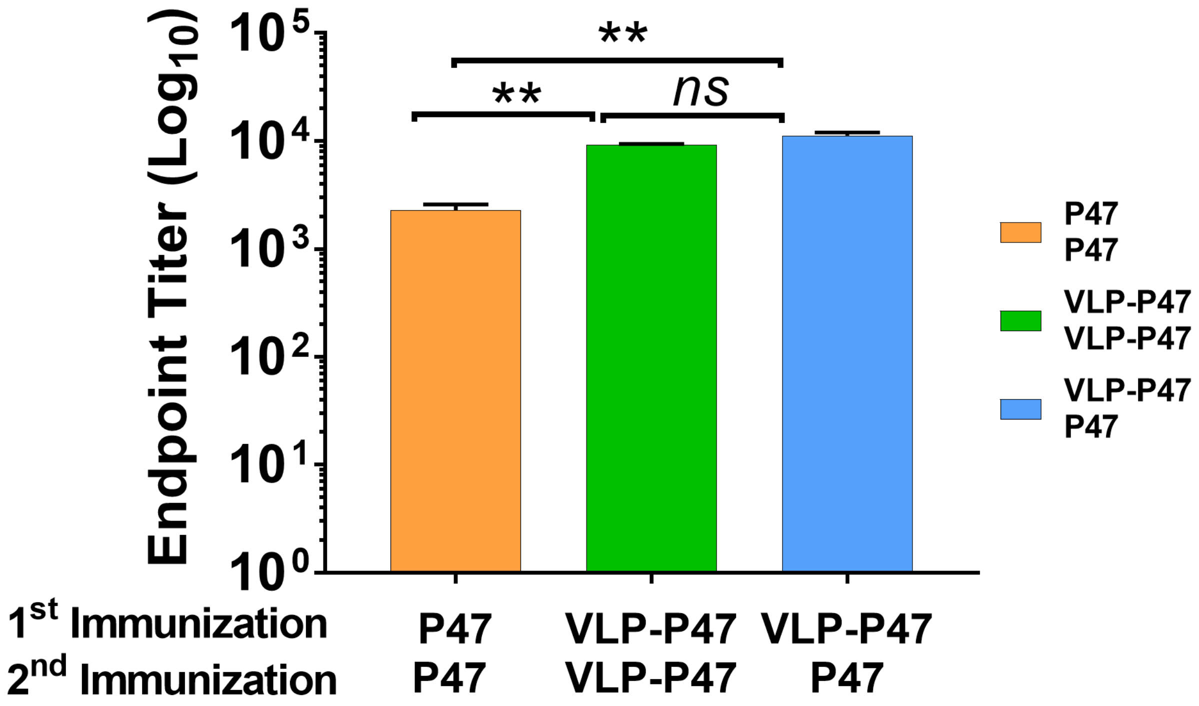

Figure S5. Immunoreactivity of purified antibodies

Sera obtained from the three immunization groups were purified to obtain antibodies. The titer of the antibodies was measured by ELISA. Column and errors bars represent mean titer ± standard deviation of two biological replicate assays. ns=non-significant, $p > 0.05$; $^{**}p \leq 0.01$.

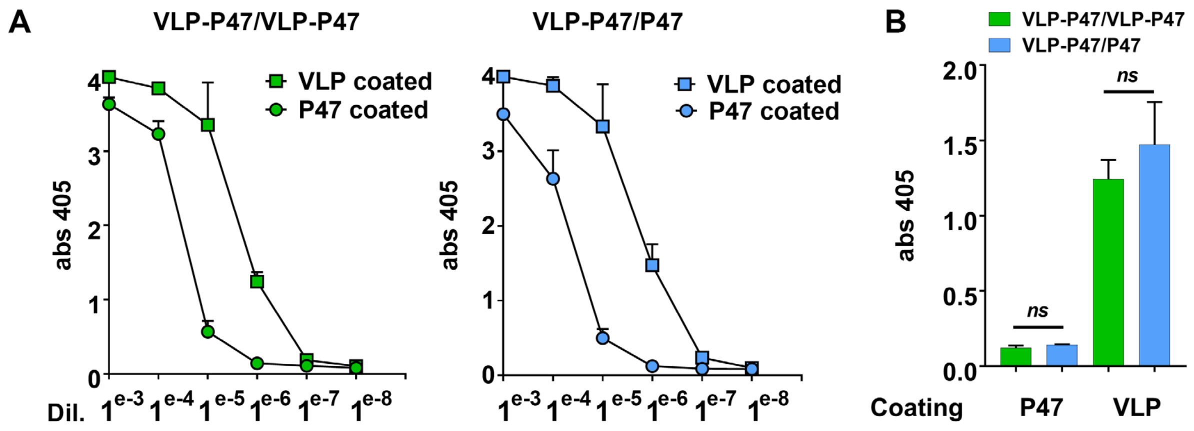

Figure S6. IgG response to P47 and VLP.

(A) Binding curves were obtained by performing ELISA with serial dilutions (10-fold) of IgG obtained from mice immunized with VLP-P47/VLP-P47 or VLP-P47/P47 onto either P47 or VLP coated plates. (B) IgG ELISA reactivity of VLP-P47/VLP-P47 or VLP-P47/P47 measured using a 1^{e-6} IgG dilution against P47 and VLP. Dots, column and errors bars represent mean absorbance value ± standard deviation of two biological replicate assays. ns=non-significant, $p > 0.05$.

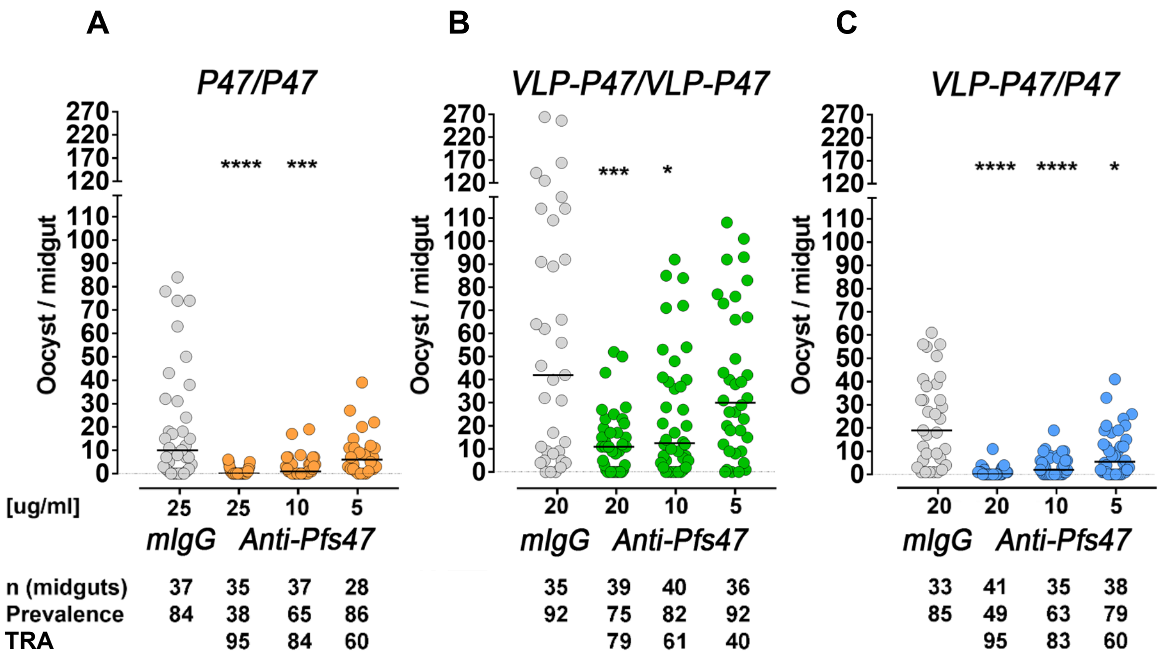

Figure S7. **Transmission reducing activities of purified mouse polyclonal IgG obtained after immunization with three vaccine preparations at decreasing concentrations, tested by a Standard Membrane Feeding Assay (SMFA).**

Dots represent the number of oocysts in individual mosquitoes and lines indicate the medians. Number of mosquitoes dissected (n); Infection prevalence (prevalence); transmission reducing activity (TRA) as percent inhibition of infection intensity in an SMFA relative to IgG control purified from naïve mice (mIgG). Medians were compared using the Mann–Whitney test: *p < 0.05; ***p < 0.001; ****p < 0.0001.)

		Salt (NaCl) concentration	
		0 mM	150 mM
pH	4.5	Stable at 200 ug/ml	Stable at 200 ug/ml
	6.5	Unstable	Unstable
	7.5	Unstable	Unstable
	8.5	Unstable	Stable at 100 ug/ml

Table S1. Effects of pH and Salt concentration on the stability of P47 measured a 4 degree.

Supplementary Methods

ELISAs

Flat-bottom 96-well ELISA plates (Immunolon 4; VWR cat # 62402-959) were coated with 100 ng/well of recombinant protein diluted in coating buffer (15 mM Na2CO3, 35 mM NaHCO3, pH 9.6) overnight at 4°C. Plates were washed three times with TBST and blocked with general block ELISA blocking buffer (ImmunoChemistry cat # 640) for 2 hrs at 37°C. Animal sera were diluted in a 1:1 ratio buffer containing blocking buffer and TBST, added to the antigen-coated wells and incubated for 2 hrs at 37°C. The plates were then washed and incubated with goat anti-mouse or anti-rabbit immunoglobulin G conjugated to alkaline phosphatase (Seracare cat # 5220-0303) secondary antibodies in 1:2500 dilutions for 2 hrs at 37°C. The plates were washed again and detection was performed using 100 µL/well of p-nitrophenyl disodium phosphate solution (Sigma 104 phosphatase substrate; 1 tablet per 5 ml of coating buffer). After 20 min, absorbance was read at 405 nm with VersaMax ELISA plate reader.

For avidity ELISA, a 15-minutes incubation step with (0, 1, 2, 4, 6, 8) M Urea was performed between primary and secondary antibody incubation steps. The relative levels of bound antibody were determined using standard ELISA procedure. The binding avidity index was calculated as the result of absorbance of wells washed with TBST-Urea (U^{+}) divided by the absorbance of wells washed with PBST (U^{-}) and multiplied by 100.

Immunofluorescence Assay (IFA)

Immunofluorescence was performed on gametocyte fixed with paraformaldehyde (PFA) as described previously[10]. The slides were washed three times in PBS for 5 min per wash, blocked and permeabilized in 5% BSA, 0.1% triton (in PBS) prior to antibody staining. The slides were overnight at 4 °C overnight with serum collected from immunized mice or naïve control diluted 1:100. Secondary Alexa Fluor 488 goat anti-mouse (Abcam ab150105) antibodies were used at 1:500 dilution and counter-stained with DAPI at 1:1000. The slides were examined using a confocal microscope at 63× magnification.
